# Longitudinal analysis of host protein serum signatures of treatment and recovery in pulmonary tuberculosis

**DOI:** 10.1371/journal.pone.0294603

**Published:** 2024-02-29

**Authors:** Samantha M. Powell, Leah G. Jarsberg, Erin L. M. Zionce, Lindsey N. Anderson, Marina A. Gritsenko, Payam Nahid, Jon M. Jacobs

**Affiliations:** 1 Biologcal Sciences Division, Pacific Northwest National Laboratory, Richland, Washington, United States of America; 2 Division of Pulmonary and Critical Care Medicine, University of California, San Francisco, San Francisco, California, United States of America; 3 Earth Systems Science Division, Pacific Northwest National Laboratory, Richland, Washington, United States of America; 4 Environmental Molecular Sciences Laboratory, Pacific Northwest National Laboratory, Richland, Washington, United States of America; The First Hospital of Jilin University, CHINA

## Abstract

**Background:**

A better understanding of treatment progression and recovery in pulmonary tuberculosis (TB) infectious disease is crucial. This study analyzed longitudinal serum samples from pulmonary TB patients undergoing interventional treatment to identify surrogate markers for TB-related outcomes.

**Methods:**

Serum that was collected at baseline and 8, 17, 26, and 52 weeks from 30 TB patients experiencing durable cure were evaluated and compared using a sensitive LC-MS/MS proteomic platform for the detection and quantification of differential host protein signatures relative to timepoint. The global proteome signature was analyzed for statistical differences across the time course and between disease severity and treatment groups.

**Results:**

A total of 676 proteins showed differential expression in the serum over these timepoints relative to baseline. Comparisons to understand serum protein dynamics at 8 weeks, treatment endpoints at 17 and 26 weeks, and post-treatment at 52 weeks were performed. The largest protein abundance changes were observed at 8 weeks as the initial effects of antibiotic treatment strongly impacted inflammatory and immune modulated responses. However, the largest number of proteome changes was observed at the end of treatment time points 17 and 26 weeks respectively. Post-treatment 52-week results showed an abatement of differential proteome signatures from end of treatment, though interestingly those proteins uniquely significant at post-treatment were almost exclusively downregulated. Patients were additionally stratified based upon disease severity and compared across all timepoints, identifying 461 discriminating proteome signatures. These proteome signatures collapsed into discrete expression profiles with distinct pathways across immune activation and signaling, hemostasis, and metabolism annotations. Insulin-like growth factor (IGF) and Integrin signaling maintained a severity signature through 52 weeks, implying an intrinsic disease severity signature well into the post-treatment timeframe.

**Conclusion:**

Previous proteome studies have primarily focused on the 8-week timepoint in relation to culture conversion status. While this study confirms previous observations, it also highlights some differences. The inclusion of additional end of treatment and post-treatment time points offers a more comprehensive assessment of treatment progression within the serum proteome. Examining the expression dynamics at these later time periods will help in the investigation of relapse patients and has provided indicative markers of response and recovery.

## Introduction

Before COVID-19, tuberculosis (TB) was the leading cause of death from a single infectious agent [1, 2]. TB is widespread and the World Health Organization (WHO) estimates 25% of the world population is infected with TB and therefore represents a large burden on the world’s population and healthcare systems [2]. An estimated 10.6 million people fell ill with TB in 2021 according to the World Health Organization’s 2022 Global TB report, with increased cases and deaths attributed in part to disruptions in care caused by the COVID-19 pandemic [3]. The onset of the COVID-19 pandemic has created drastic setbacks, heightening the importance to refocus efforts on improving tools for prevention, diagnosis, and treatment of TB.

The identification of non-sputum based surrogate markers of efficacy will accelerate hampered tuberculosis (TB) drug development. The current standard for use as a surrogate endpoint in phase 2 studies remains focused on culture conversion and the most studied is the two-month culture status which exhibits low sensitivity and modest specificity for predicting outcomes after treatment completion [4–7]. Additionally, being dependent on sputum as the sole method of measure, especially culture-based monitoring, can be challenging in extrapulmonary TB and in patients with paucibacillary disease as seen in HIV-coinfected patients and children [8, 9]. Both low sputum volume and collection quality decreases in response to treatment as many patients cannot provide sputum samples for culture after a few weeks of treatment. The development of non-sputum-based proteome biomarkers for measuring treatment response would represent an advance for individual monitoring of TB patients as well as serving as an intermediate marker for use in TB drug development [10]. Multiple studies have previously focused on outcomes only related to initial short-term responses to treatment (i.e., 8-week time points) [10, 11], in conjunction with various treatment efficacy endpoints (i.e., culture conversion). Considering standard treatment regimens for TB end at 26 weeks, there is a strong rationale to investigate more thoroughly across the entire treatment period and into post-treatment response and recovery.

Here we present a study focused on advancing a general understanding of the longer-term serum proteomic signatures of TB patients in response to interventional treatments. Our study population includes patients exclusively from South Africa and longitudinal sample collection of five timepoints from baseline to 52 weeks. Our goal was to build upon the short-term host treatment responses that have been previously reported [11] combined with end of treatment and post-treatment serum plasma signatures for analysis.

## Materials and methods

### Ethics statement

Written informed consent was obtained from all study participants for collection of serum for TB-related research. The institutional review board at University of California, San Francisco approved this biomarker study (UCSF IRB Number: 12–10360).

### Study population

The parent study was MARK-TB (Markedly Accelerating Research with Knowledge of Tuberculosis Biomarkers study https://www.mark-tb.org) which included participants with durable cure 12 months post-treatment completion from the Phase 3 REMoxTB trial and the National Tuberculosis Program at the same sites at which REMoxTB was conducted [12]. All participants in this trial received standard rifampin doses. The standard of care control study arm required 26 weeks of treatment whereas the experimental treatment regimens were 17 weeks in duration. Blood serum for protein analysis was collected at pretreatment as a baseline and at weeks 2, 4, 8, 17, 26, and 52 post treatment. Time point collections for this analysis utilized baseline, 8, 17, 26, and 52 weeks for REMoxTB participants and at baseline, 4, 8, 26, and 52 weeks for National Tuberculosis Program (NTP) participants. All samples were collected in 2013–2014.

### Selection of participants

For this study 30 cured patients were randomly selected from the MARK TB data and biospecimen repository. Detailed demographic, clinical, radiographic and microbiologic data were collected using standardized data forms as part of the parent clinical trial [12]. No authors had access to patient identifying information.

### Sample processing

Serum samples were collected at baseline (t = 0) and at weeks 8, 17, 26 and 52. The samples were processed as previously described [11, 13]. Briefly, the individual serum samples were then partitioned and depleted of 14 specific highly abundant proteins using a ProteomeLab^™^ 12.7×79.0-mm human IgY14 LC10 affinity LC column (Beckman Coulter, Fullerton, CA). The depletion flow-through was further processed by automated protein isolation, denaturation, tryptic digestion, and peptide isolation as previously described [13]. Tandem Mass Tag (TMT) 11 labeling (ThermoFisher Scientific) was performed as indicated by the manufactures protocol. 50 ug of peptide were included in each channel, two patients per plex, and inclusion of a universal reference peptide standard for each TMT experiment across 11 channels (TMT11). Each TMT11 multiplex experiment was subjected to off-line HPLC separation as previously described [14, 15] resulting in a concatenated 12 fractions for each TMT11 experiment.

### MS analysis

A PAL autosampler (CTC Analytics AG, Switzerland) equipped with two six-port valves (Valco Instruments Co. Inc., Houston, TX) was used for sample injection. After being injected into a 25-μL loop, the sample was concentrated into an SPE column (150 μm i.d., 360 μm o.d., 4 cm long) at a flow rate of 5 μL/min. The nanoLC column (50 μm i.d., 360 μm o.d., 50 cm length) was packed with 3-μm C18 packing material (300-Å pore size, Phenomenex, Terrence, CA). Mobile phases (Buffer A: 0.1% formic acid in water; Buffer B: 0.1% formic acid in acetonitrile) were delivered by a nanoUPLC pump (Dionex UltiMate NCP-3200RS, Thermo Scientific, Waltham, MA) at a flow rate of 150 nL/min. The LC method was programmed as a 100-min linear gradient from 8% to 22% Buffer B followed by a 15-min linear gradient to 45% Buffer B, after which the column was washed with 90% Buffer B for 5 min and re-equilibrated with 2% Buffer B for 20 min. The separated peptides were analyzed using a Thermo Scientific^™^ Q Exactive^™^ Plus. Data were acquired in a data-dependent mode with a full MS scan from m/z 350–1800 at a resolution of 70,000 at m/z 400 with AGC setting set to 5×10^5^ and maximum ion injection period set to 50 ms. Top 10 precursor ions having intensities >2.5×10^4^ and charges between +2 and +8 were selected with an isolation window of 2 Da for MS/MS sequencing at an HCD energy of 33%. MS/MS spectra were acquired at a resolution of 17,500. The AGC target was 1×10^5^ and the maximum ion accumulation time was 200 ms using a dynamic exclusion time window of 40 s.

### Data processing and analysis

LC-MS/MS raw data were converted into data files using Bioworks Cluster 3.2 (Thermo Fisher Scientific, Cambridge, MA, USA). The MSGF+ algorithm [16] was used to search MS/MS spectra against the Human Uniprot database (Uniprot_SPROT 2017-04-12; 20,198 sequence annotation entries). The key search parameters used were 20 ppm tolerance for precursor ion masses, +2.5 Da and -1.5 Da window on fragment ion mass tolerances, no limit on missed cleavages, partial tryptic search, no exclusion of contaminants, dynamic oxidation of methionine (15.9949 Da), static IAA alkylation on cysteine (57.0215 Da), and static TMT modification of lysine and N-termini (+144.1021 Da). The decoy database searching methodology [17, 18] was used to control the false discovery rate at the unique peptide level to <0.01% and subsequent protein level to <0.1%. Quantification was based upon initially summing to the protein level the sample specific peptide reporter ion intensities captured for each channel across all 12 analytical fractions. Final data for statistical analysis was the ratioing of each protein summed value with the pooled reference control within each TMT11 experiment to adjust for experiment specific variability. The mass spectrometry proteomics data have been deposited to the ProteomeXchange Consortium [19] via the PRIDE partner repository with the dataset identifier PXD040546 and MassIVE Accession MSV000091392.

Statistical analysis was performed using an in-house software, Inferno [20]. Data first underwent normalization transformation (log2) followed by stratification upon disease severity and time of serum collection to identify discriminatory protein/pathway signatures. Significant proteins were identified as having a reported p-value<0.05 (ANOVA), with >90% observational coverage across all time points and patients. This approach was utilized to obtain timepoint specific comparisons for direct correlation with previous results and outcomes as compared to other analysis approaches. Pathway enrichment was performed using Reactome [21] and mapped with Morpheus [22] and ggplot2 [23].

## Results

### Study population and identified proteins

**Fig 1:**
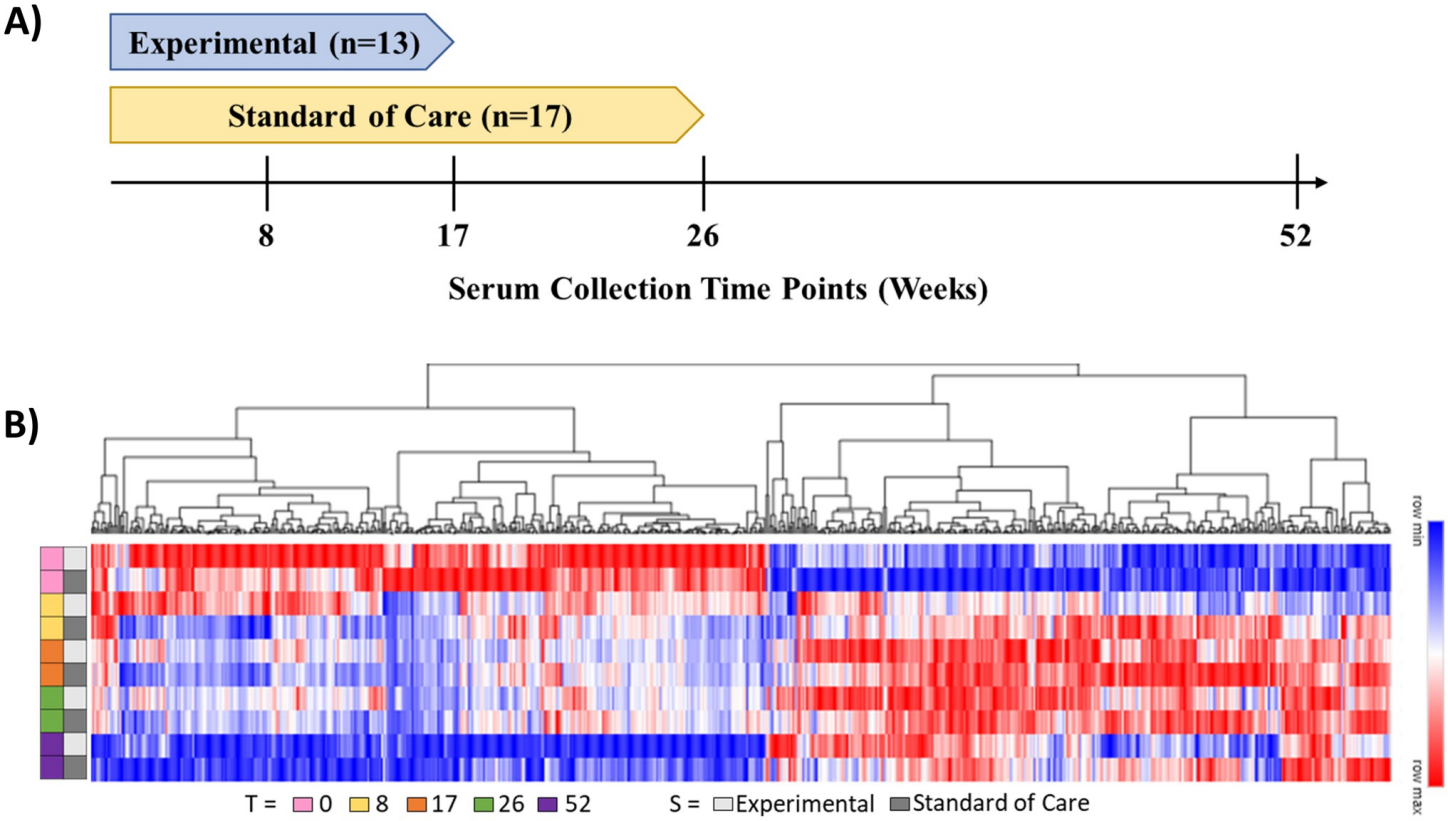
Overview of the serum proteome study. (A) Timeline of the different patient population serum collection timepoints relative to treatment arm for a total of 30 patients. (B) Hierarchical clustering heatmap of the composite 676 proteins which exhibited differential abundance within the measured grouped timepoints, stratified by treatment type. Red to blue color gradient corresponds to higher to lower relative protein abundance values.

A breakdown of the study is shown in Fig 1A and Table 1 details the clinical metadata of the 30 participants, whose serum at longitudinal collections 0, 8, 17, 26, and 52 weeks was used for this study. Utilizing a sensitive LC-MS based analytical platform, see Materials and methods for details, a total of 1889 proteins were identified and quantified across this patient sample set for further analysis (**S1 Table in** S1 File). Of the 1889 identified proteins, 676 had significant abundance differences as indicated by >90% coverage across all patients and time points as well as a measured p<0.05 for at least one time point comparison with baseline (week 0) (see Fig 1B and **S2 Table in** S1 File).

**Table 1:** Characteristics of study participants.

Category	No. of Patients (%)
All Subjects	30 (100)
Female	12 (40)
Age	
<30	16 (53)
30–60	14 (47)
Underweight (BMI < 18.5)	13 (43.3)
HIV Positive	0 (0)
Cavity in baseline Chest X-Ray	24 (80)
Sputum Smear Category	
≤1	16 (53)
2–3+	14 (47)
Treatment Type	
Standard of Care	17 (56.7)
Experimental	13 (43.3)

### Extension of analysis through post-treatment

**Fig 2:**
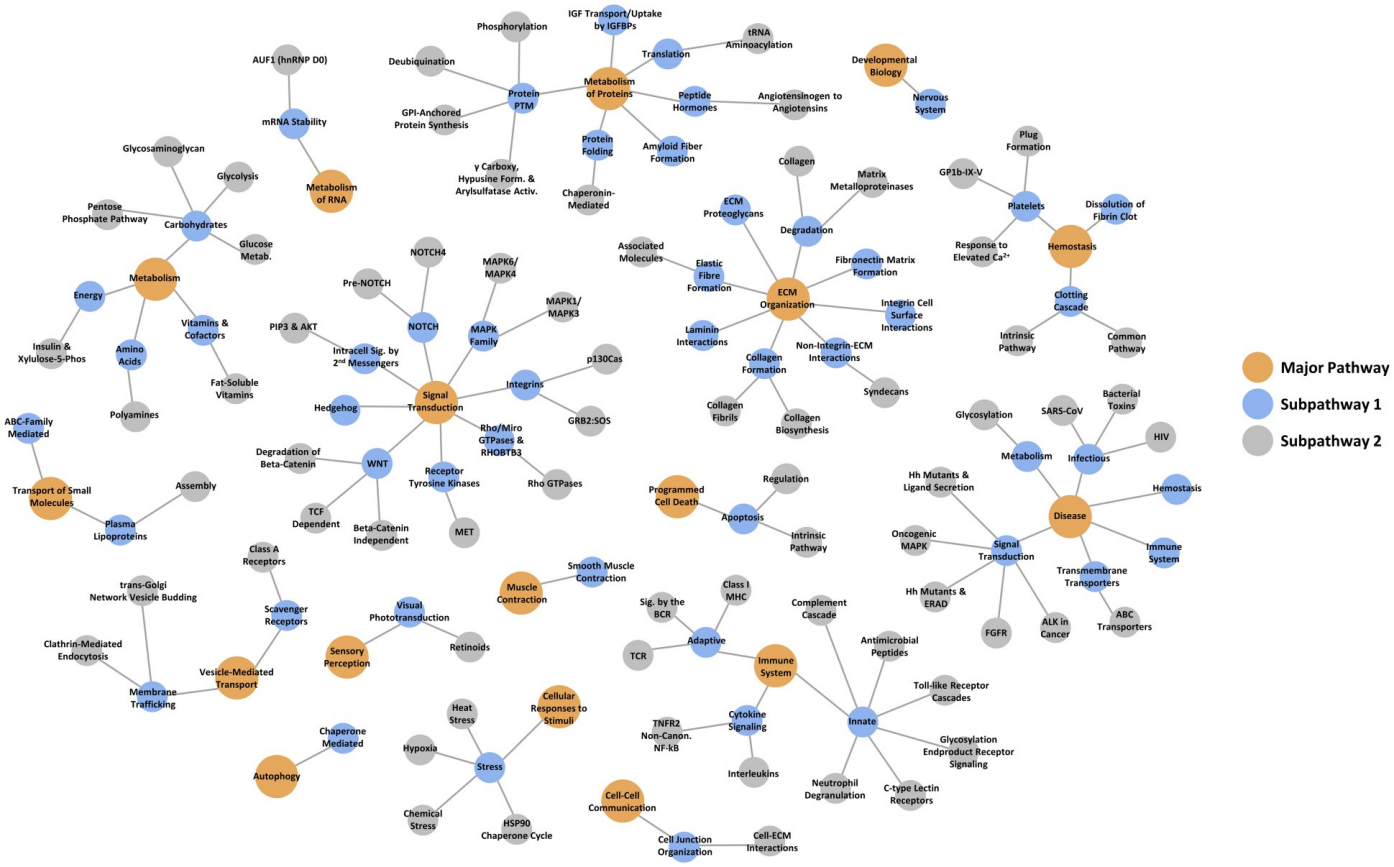
Pathway mapping of the proteome signature. Differentially abundant serum proteins were pathway mapped across the longitudinal collection of baseline through 52 weeks. Hierarchical categorization is denoted by color and size.

As previous studies have primarily focused on biomarker changes over the initial 8 weeks of TB treatment, we expanded our differential serum protein expression analysis through end of treatment and included time points following the completion of treatment. The comparisons made at each time point relative to baseline (t = 0) control, resulted in the 676 proteins identified as differentially changing in relative abundance over time (**S2 Table in** S1 File). In total, these proteins mapped across a broad variety of critical pathways (Fig 2) with the top pathways encompassing the core regulatory immune system (esp. innate immune system and neutrophil degranulation, cytokine signaling), hemostasis (esp. platelet activation, signaling and aggregation), vesicle-mediated transport, and extracellular matrix organization. See **S3 Table in** S1 File for the full network mapping details.

**Fig 3:**
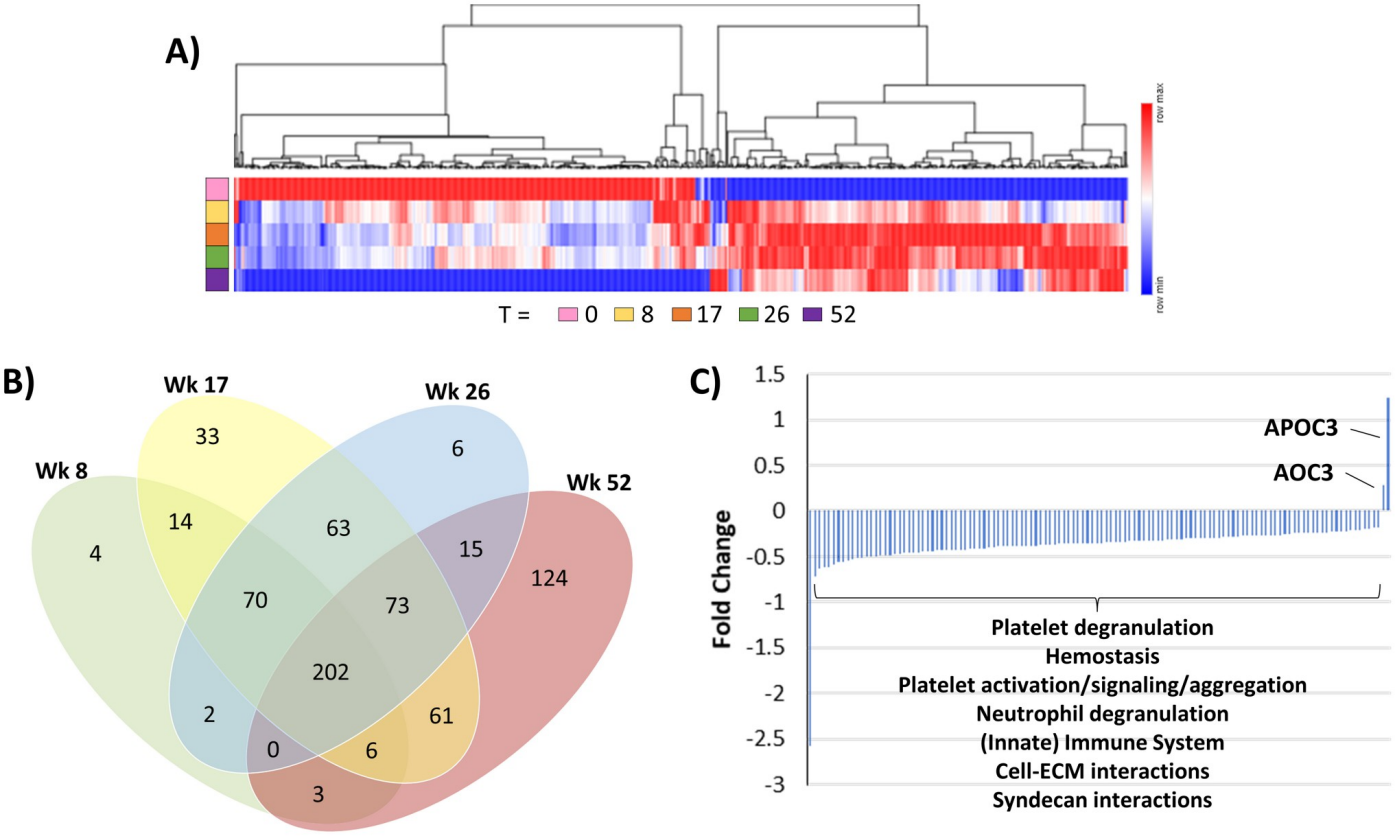
Longitudinal characterization of serum proteome signature. (A) Changes across time of the 676 significant proteins, (B) the distribution of the significant proteins by time point, and (C) the fold changes of the 124 unique proteins at 52 weeks (See S6 Table in S1 File for more detail).

Observations across the full 52-week timeline, as seen in Fig 3A, revealed a clear differentiation between the proteins upregulated (red) or downregulated (blue) at baseline relative to other timepoints. Baseline upregulated proteins consistently trended downwards through treatment and then strongly into post-treatment at week 52 (blue). However, in general downregulated proteins at baseline (blue), rebound highest during treatment, peaking at 17 weeks, followed by attenuation by week 52. A similar trend is observed for the total number of significant proteins at each time point. By 8 weeks, 301 proteins were observed as significant, increasing to 522 by week 17, and then reducing back to 431 by week 26. Finally, a slight increase is observed reaching 484 significant proteins by week 52 (See **S5 Table in** S1 File for the list of significant proteins at each time point). This trend is again observed by looking at the number of unique differential proteins identified within each time point group (Fig 3B). Although, fewer unique significant proteins are observed at week 8 (4 proteins), i.e., the majority of differential proteins identified at week 8 remained differentially abundant throughout the time course with a brief uptick in the number of unique proteins found by week 17 (33 proteins). By week 26 (6 proteins) a significant jump in the number of unique proteins found by week 52 (124 proteins). As there was an interest to determine what signatures were best represented during post-treatment recovery, we looked specifically at proteins uniquely expressed at 52 weeks. For 124 proteins denoted in Fig 3B, all but two had a reduction in abundance by week 52 relative to baseline observations, see Fig 3C and **S6 Table in** S1 File.

### Analysis by treatment

Participants in this study were part of either the REMoxTB clinical trial or the National TB Program of Cape Town, South Africa (NTP) and underwent one of three treatment types—standard of care, or one of two experimental clinical trial treatments, as described previously, with no major differences between the two experimental treatments [12]. Therefore, participants were grouped into either experimental (n = 13) or standard of care (n = 17).

A large overlap was observed between the two treatments in which pathway specific proteins were influenced over time (Fig 1B). Previous studies have defined a consistent set of serum proteins (TTHY, AFAM, CRP, RET4, SAA1, PGRP2) with an observed differential response to experimental treatment type after 8 weeks [11]. **S1 Fig in** S2 File shows the current cohort’s changes in this same set of serum proteins across 52 weeks. Although the change across the 52 weeks is significant, any major difference in the two treatment types detected at each respective 17- and 26-week timepoint were deemed inconclusive.

### Analysis by severity

The TB severity of patients was designated based on their smear grade [24]. Within the present study, patients were grouped into two different severity groups–mild (smear grade <2; n = 14) or moderate to severe (smear grade ≥2; n = 16). For this comparison, severity, as well as time points, were utilized as variables in a mixed effect model, and a total of 461 proteins were identified as significantly changing over time based upon severity (**S7 Table in** S1 File).

**Fig 4:**
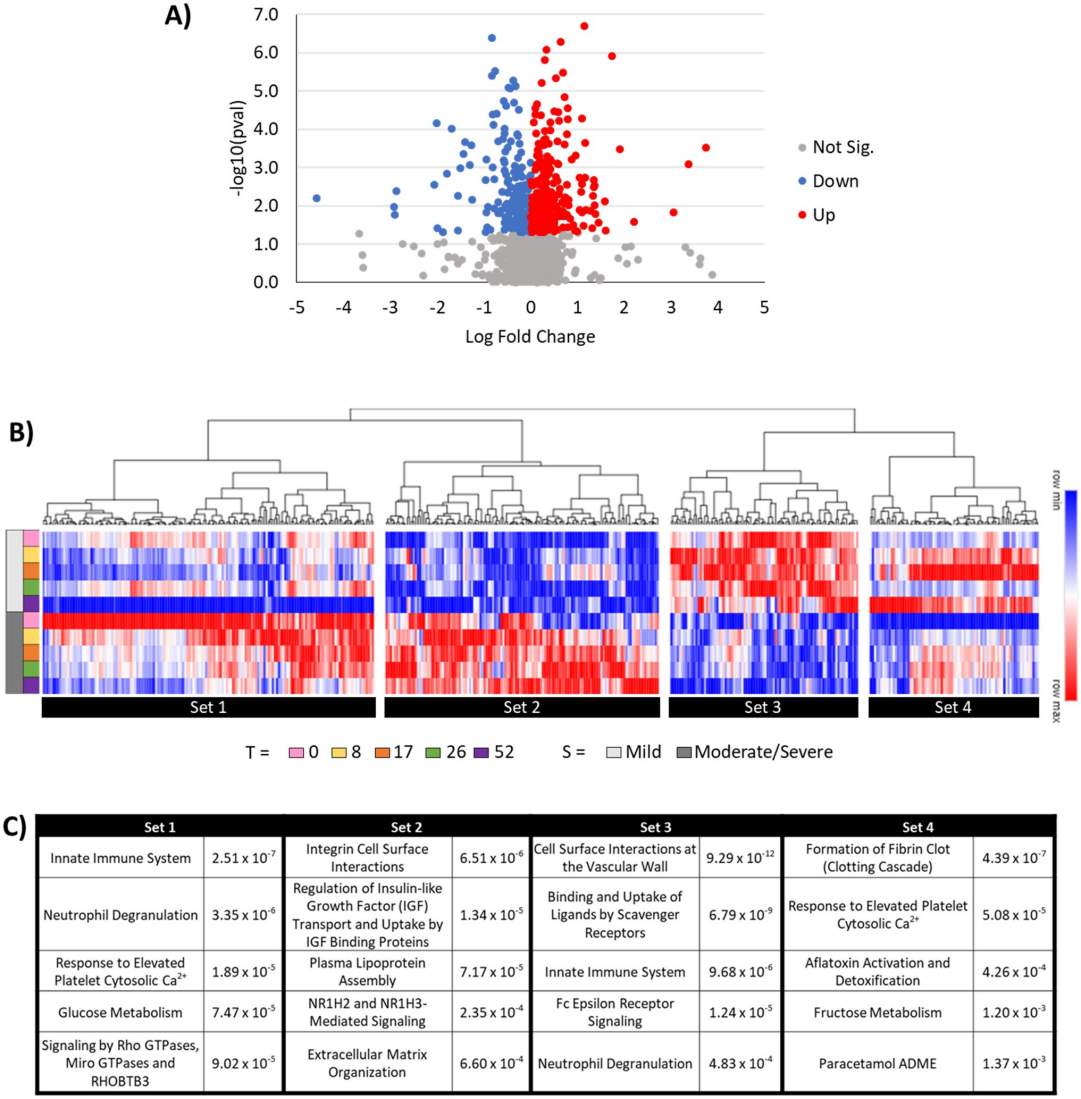
Disease severity proteome signature characterization. A total of 461 proteins were identified as significant across time and disease severity. (A) At time zero, there’s an even distribution of proteins that were up or downregulated between the moderate to severe group and the mild group (See S1 Fig in S2 File for other time points). (B) Differences between the moderate to severe group and the mild group are mapped across time and split by severity level, resulting in four major sets according to initial protein abundance and direction of fold-change. (C) Top 5 significant non-redundant Reactome pathway mappings, and respective p-value, for each of the four sets (refer to S3 Table in S1 File for Reactome pathway identifiers).

Clear differences were observed between the mild and moderate to severe groups (Fig 4). While these differences are especially evident at time zero (Fig 4A), the differences still held true across the full 52 weeks (Fig 4B and **S2 Fig in** S2 File). The proteins identified could be separated into four sets based on their initial abundance values and directionality of fold-change (See Fig 4B for set references and **S3 Table in** S1 File for Reactome pathway identifiers). In the first set, these proteins had much higher abundance in the moderate to severe group than in the mild group, but the overall abundance in both groups decreased over time with treatment. Set 1 is driven by acute phase reactants, immune cell activations to infection, and various cell mediated signaling. In the second and third sets, there were no changes in the protein abundance across time, but they were consistently higher in the severe to moderate group (Set 2 driven by cell surface markers, IGFBP regulation, and lipoprotein markers) or consistently higher in the mild group (Set 3 driven by an immunoglobulin responses, hemostasis, and additional neutrophil granulation). In the fourth set, the protein abundance was initially higher in the mild group, and the abundance increases in both groups across time. Set 4 is driven by various coagulation factors, plasminogen, and other hemostasis proteins. Reactome pathway mapping of each subset reveals a distinct protein signature of differentiation based upon severity (See Fig 4C).

## Discussion

The host response to treatment of acute pulmonary TB is a dynamic temporal process which we have investigated longitudinally through proteomic analysis of serum at specific timepoints. This complemented previous studies focused on early timepoints, i.e., 8 weeks of treatment, to correlate culture status with prospective serum markers of treatment efficacy/failure [11]. In this study, we moved beyond 8 weeks and captured the serum proteome signal at end of treatment, 17 and 26 weeks, and post treatment at 52 weeks. Each quantitative timepoint comparison provided insight into the mechanics of the proteome host response during each of these phases of treatment and recovery.

Interestingly, our study showed that almost all proteome changes at 8 weeks were retained through end of treatment and beyond through 52 weeks, though later time points had more proteins identified as significant (Fig 3B). This would be expected as TB patients complete recovery through the long treatment regimen; it would be anticipated that their host proteome would continue to diverge from the acute infective state at timepoint 0.

**Fig 5:**
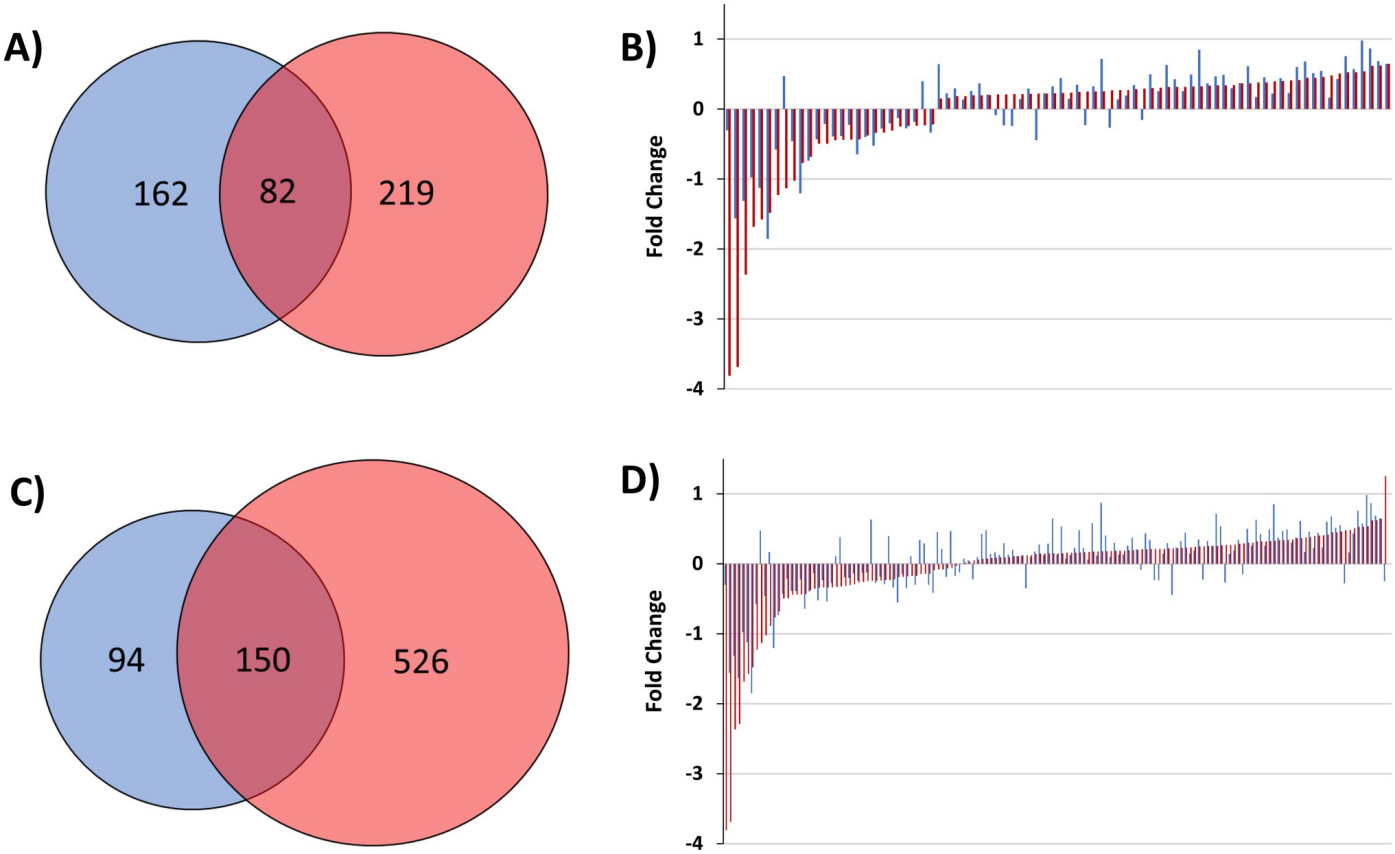
Overlap with previous TB treatment studies. Comparison of a previous 8-week study identifying 244 significant proteins [11] (blue), with the 8-week time point (A-B) and overall 52-week time course (C-D) of the present study (red), identifying 301 and 676 significant proteins, respectively. (A) 82 total proteins overlap between the previous and present studies at the 8-week time point, and (C) 150 total proteins overlap across the full 52-weeks. The respective fold-changes of the (B) 82 and (D) 150 overlapping proteins show that the directionality of the changes is consistent across most proteins.

Previous comprehensive proteome studies compared 289 patient serum signatures at baseline and at eight weeks of TB treatment which identified 244 significantly changing protein signatures [10, 11]. Of these, 82 proteins directly overlap between the previous and present study at the 8-week time point (Fig 5A). Comparing the previous study to the full 52-week study performed here, 150 proteins overlap (Fig 5C). Overall, however, the specific 8-week time point comparison of the overlapping 82 proteins at the same point, or the 150 proteins across the full 52-week study, showed extensive congruence between the two studies based upon the overlapping proteins’ correlation with biological pathways. Modulated serum proteins corresponded to the immune system (esp. complement cascade, inflammation, defense), lipid transport and metabolism, proteolysis and apoptosis, coagulation cascade and hemostasis, and cell adhesion and extracellular matrix (Fig 2).

The observed protein specific fold changes (Fig 5B and 5D) identified only a few exceptions to the overall consistency in directionality of the fold-change between the studies (**S4 Table in** S1 File), which helped drive similar pathway mapping between the two studies. At the 8-week time point, there are nine proteins of the 82 that change in opposite directions, and this number increases to 22 by the 150 by the end of the full 52-week study. However, where directionality differed was notable. Protein SAA4, a constitutively expressed yet acute phase protein was previously upregulated in the 2018 study compared to its strong downregulation at week 8 in the present study. It is likely that its dual role as a member of HDL contributes to this discrepancy [25]. The major difference between the 2018 study and the current study is the distribution of patients where the 2018 study contains patients across both western countries and Africa, where the current study only contains African patients. The rebound in lipid transport and hence HDL components due to treatment at 8 weeks was largely significant in the more diverse patient population, likely due to inclusion of western nutritional effects [10], which appears more muted in African-only populations. This is consistent with the presence of other lipid transport proteins RARR2 and APOC3 which were identified as significantly upregulated at week 8 in the 2018 study but are seen as slightly downregulated in the current African-only study. Compared to the previous 8-week only studies, in the present study, more proteins were found as significant in later timepoint comparisons, however the pathways that these proteins mapped to, remained consistent, even at the final 52-week time point. Overall, these observations are consistent with the previous study, and provide a foundation for the observed quantitative trends observed through 52 weeks. The largest proteome changes observed across the time course appeared at the 17- and 26-week time points indicating that these time points appear to be pivotal to monitor for TB disease and treatment progression (Fig 3B). Representing treatment endpoints, it is at these points where treatment was at its full final effect, possibly driving larger changes in protein signatures. Little is known concerning the direct host serum protein expression after long-term antibiotic treatment. We certainly see a continuation of TB disease resolution at these treatment endpoints (Fig 3B, **S1 Fig in** S2 File), yet not all changes continue into week 52 (see the unique set of 124 differentially abundant proteins at week 52). It is likely that these mid-timepoint signatures concurrently reflect a direct host-response to treatment beyond disease resolution, though to decouple these effects is difficult. Observing those protein signatures which are consistent across weeks 17 and 26, yet do not remain significant into week 52, (Fig 3B, **S5 Table in** S1 File, 70 + 63 for a total of 133 proteins), we observe an increase in abundance for almost all 133, compared to time point zero. The largest fold-change observed was SHBG, which abundance is known to be directly affected by rifamycin treatments [26], and has been identified as a disease progression biomarker by others [27]. Overall, the surge of up-regulated signature is replete with cell adhesion molecules (BCAM, ICAM3, CD166, CD44, CADM1, PECA1) as well as receptor signaling (NOTC4, NOTC3, IGF1, CSF1R, MET, IL6RB, BMP1). Overall, there appears a broad and sustained cellular activation until end of treatment, which mitigates by 52 weeks. This has implications concerning length of treatment and instances of relapse. Gaining a deeper understanding of the host response to treatment and its subsequent off-ramping will help inform the mechanisms involved in mitigating relapse.

Though treatment regimens ended at either 17 or 26 weeks, as discussed, large changes were still occurring post-treatment out to 52 weeks (Fig 3), as there were 124 uniquely significant proteins identified at the 52-week timepoint (Fig 3C, **S6 Table in** S1 File). This extended post-treatment timeframe response in the plasma implies an extended and continuing period of recovery, and/or decoupling of the host response to treatment, well beyond last timepoint of treatment. Pathway analysis of the 122 down-regulated proteins, Fig 3C, confirms the continued attenuation of the cellular immune responses towards basal recovery levels. Of additional interest are the two proteins observed uniquely upregulated at 52 weeks, apolipoprotein C3 (APOC3) and membrane primary amine oxidase (AOC3). APOC3 is one of the many apolipoproteins upregulated upon treatment, where all others have primarily rebounded at the earlier timepoints, APOC3 appears unchanged until 26 weeks, and not significantly changed until 52 weeks (**S2 Table in** S1 File). AOC3 or VAP-1, is reported to have multiple functions, with roles in inflammation facilitating transmigration of leukocytes [28] as well as a role in adipogenesis [29]. Based upon AOC3’s correlative abundance changes with circulating lipid transport proteins, it likely represents another late-stage recovery marker of lipid metabolism.

Understanding the role of disease severity in the context of treatment and recovery has important implications in the stratification and management of TB patients. Four distinct clusters of protein expressions were observed from the 461 protein signatures deemed significant across the time course when comparing between disease severities (Fig 4B). Each represented a unique proteome signature depending upon its progression over time (Fig 4C). Of note is that two clusters remain largely consistent from 0 weeks to 52 weeks (set 2 and set 3, Fig 4B), potentially implying that patient protein signatures of severity persist until well into recovery, and at 52 weeks, there is still a differentiating signature of initial disease severity. This intrinsic stratification could be driven by multiple host and/or disease factors, and with the limited patient population of n = 30, it is difficult to surmise the core drivers and if week 52 represents a general “return to normal” phenotype or would occur at different time points based on severity of disease. To fully assess when “return to normal” occurs for patients with different disease severity, a well-matched cohort of participants with some standardized measure of disease would be required for comparison, but the current data suggests that it is beyond 52 weeks. The most significant upregulated pathways in severe patients in set 2 reflects multiple cell surface interactions and regulations (Integrin, Insulin like growth factor (ILGF) signaling, and extracellular matrix) and lipoprotein modulation with some general immune system alterations. Integrin signaling appears driven by receptors such as ITAL, VGFR2, ITB2, ITA7 and MADCA. ILGF signaling was driven by IGF1, IBP5, IBP3, FSTL3, and SPP2. Down regulated pathways in set 3 are broadly immune regulation focused, where even though these patients cleared TB bacterial infection, and overall showed improved immune response and effective treatment efficacy, there remains a remanent suppressive immune signature due to the initial severity still present at 52 weeks, i.e., CD14, CATD, CD177, ILF2, and LEAP2.

Within the present study, the focus was to longitudinally investigate patients who had recovered from TB infection with no reported incidence of relapse. We recognize that the current study does have some limitations based on the small sample size, but forms the basis for understanding a cured patient profile specific to distinct time points related to treatment and post-treatment. We were somewhat surprised at the nature of the proteome signature at 52 weeks, and the inclusion of a matched cohort would provide a more reasonable baseline to benchmark many of these protein signatures to address at what point a “return to normal” signature occurs. Possibly future sample collections could be extended beyond 52 weeks, though this might be logistically challenging at a larger scale. Of a more immediate note is the analysis of a patient cohort who have relapsed for comparison to the current non-relapsed cohorts in the hopes of identifying and validating the relevant timeframe/signature and possibly specific markers to disease relapse and progression. With this study we have generated a robust longitudinal signature of TB recovery, from which the altered mechanism reflected in the protein expression of serum can be compared in matched relapse patients. Continuing studies include analysis of matched relapse patients for comparison with the current recovered TB patient signatures, followed by targeted validation studies of the differential signatures for treatment relapse utilizing MS based selective reaction monitoring (SRM) assays.

## Supporting information

S1 File (XLSX)

S2 File (DOCX)
