# Supplementary material for: Longitudinal analysis of host protein serum signatures of treatment and recovery in pulmonary tuberculosis
Source: PLoS One. 2024 Feb 29;19(2):e0294603. doi: 10.1371/journal.pone.0294603 (PMC10903915; doi:10.1371/journal.pone.0294603)
Supplement: S2 File — (DOCX) [file pone.0294603.s002.docx]

**Supplemental Information for: Longitudinal TB Study**

Samantha M. Powell^1^, Erin L. M. Zionce^2^, Lindsey Anderson^1^, Leah Jarlsberg^3^, Marina A. Gritsenko^1^, Payam Nahid^3^, Jon M. Jacobs^4^

^1^Biologcal Sciences Division, Pacific Northwest National Laboratory, Richland, WA, USA

^2^Earth Systems Science Division, Pacific Northwest National Laboratory, Richland, WA, USA

^3^Division of Pulmonary and Critical Care Medicine, University of California San Francisco, San Francisco, CA, USA

^4^Environmental Molecular Sciences Laboratory, Pacific Northwest National Laboratory, Richland, WA, USA

**Supplemental Information Contents:**

Figure S1. Treatment types whisker plots

Figure S2. Volcano plots for severity groups

**
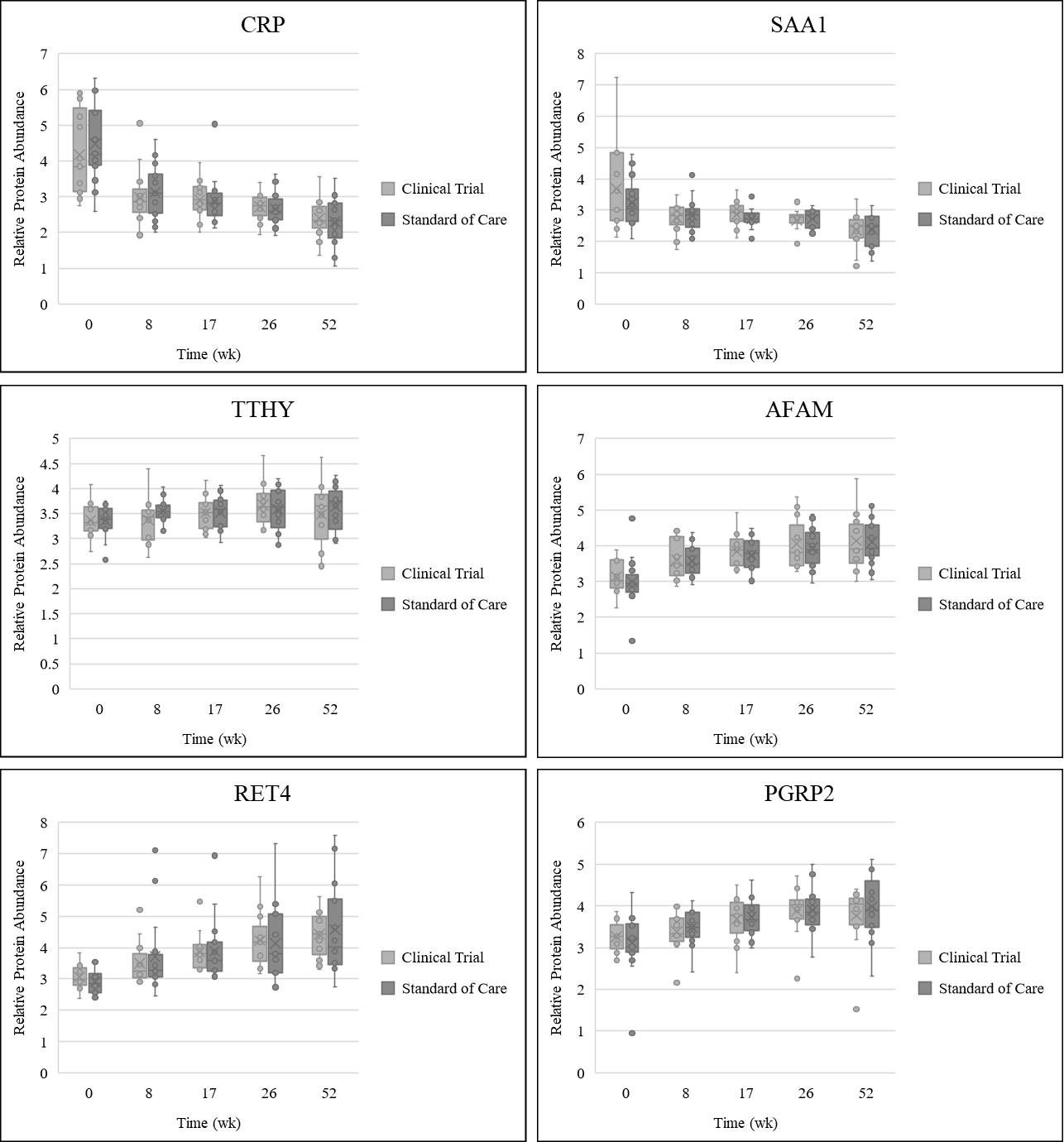
**

**Figure S1**. Change in protein levels across time points, comparing the two treatment types, clinical trial versus standard of care.


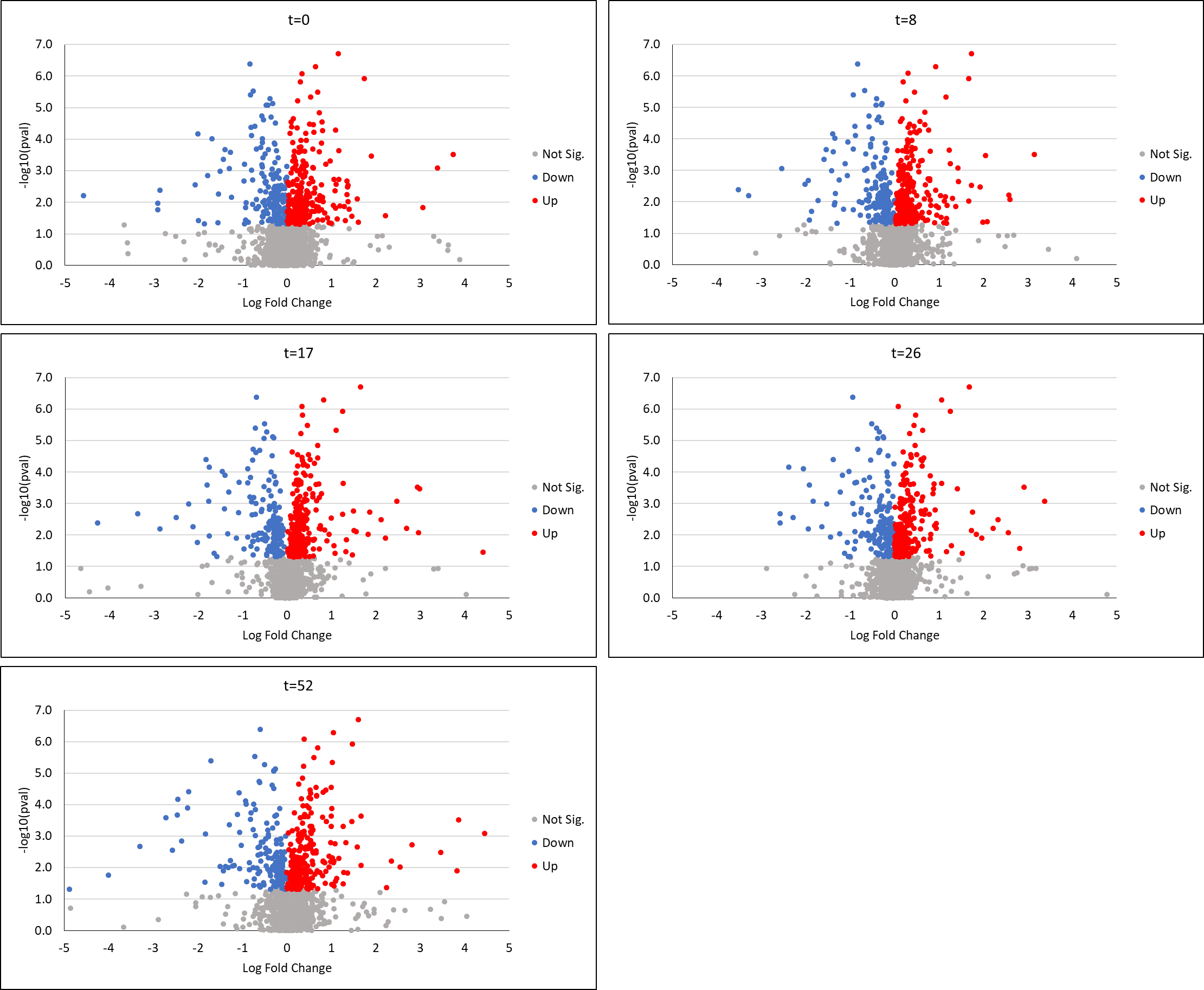


**Figure S2**. Volcano plots representing change in severity groups at each time point.
